# 78 Which Characteristics Explain Physical Activity Behaviour in Adults in the Netherlands: A Cross Sectional Study

**DOI:** 10.1093/eurpub/ckae114.123

**Published:** 2024-09-26

**Authors:** Annemarie van der Vegt, Barbara Snoeker, Tessa Schurink-van ‘t Klooster

**Affiliations:** National Institute for Public Health and the Environment (RIVM), Netherlands; National Institute for Public Health and the Environment (RIVM), Netherlands; National Institute for Public Health and the Environment (RIVM), Netherlands

## Abstract

**Purpose:**

To properly align the physical activity (PA) government policy with the Dutch population, it is necessary to gain knowledge on which personal characteristics are associated with PA behaviour. Although this association was explored for each characteristic separately in previous research from the Dutch National Institute for Public Health and the Environment (RIVM), the influence of several characteristics in one model has not been investigated. The current study aims to determine which of 15 selected characteristics explain PA behaviour of Dutch adults best, and gain additional knowledge about interactions between characteristics.

**Methods:**

Data of the Dutch Health Survey/Lifestyle monitor (Statistics Netherlands (CBS) in collaboration with RIVM) from 2018-2022 was used. In total, 36,838 Dutch adults (age≥18) were included. Both univariate and multivariable logistic regression analyses were conducted for three outcomes: adherence to Dutch PA guidelines, weekly sport participation, and weekly active transportation (walking/cycling to work/school). A selection of personal characteristics (9 demographic, 6 health-related) served as input for the regression analyses. Results of univariate and multivariable analyses were interpreted both separately and in comparison to each other.

**Results:**

Univariate results show significance for all characteristics in all three outcomes, except for gender in weekly sport participation. Multivariable, being married, having psychological complaints and a higher income are no longer significant for adherence to PA guidelines and active transportation. Adults with a higher age, higher education, normal body mass index, perceived health marked ‘very good’, without physical impairments, who are living extremely urbanised and do not smoke show high odds for all three outcomes.

**Conclusions:**

The current results provide new insights into which personal characteristics are important regarding PA behaviour of Dutch adults, regardless of other characteristics a person might have. However, the explained variance of the models was very low and only a small selection of relevant characteristics for PA behaviour was included. It is recommended to include additional information in the models (e.g., environmental and motivational factors) to further explain adult PA behaviour in The Netherlands, and to adapt PA policy accordingly.

**Support/Funding Source:**

This research was funded by the Ministry of Health, Welfare and Sports of the Netherlands.

